# Gram‐Scale Synthesis of Hafnium‐Rich Carbon Dots for Preclinical Computed Tomography Imaging Across Various Systems

**DOI:** 10.1002/advs.202517986

**Published:** 2026-01-31

**Authors:** Shuo Li, Hengrui Wu, Jianqi Deng, Qiyu Sun, Yuping Zhang, Cai Zhang, Jinbin Pan, Dingbin Liu, Xuejun Zhang, Quan Zou, Xiaoyuan Chen, Shao‐Kai Sun

**Affiliations:** ^1^ School of Medical Imaging Division of Medical Technology Tianjin Key Laboratory of Functional Imaging Tianjin Medical University Tianjin China; ^2^ Department of Radiology Tianjin Key Laboratory of Functional Imaging Tianjin Medical University General Hospital Tianjin China; ^3^ Department of Radiology Tianjin Medical University Cancer Institute and Hospital National Clinical Research Center of Cancer Tianjin's Clinical Research Center for Cancer Tianjin China; ^4^ College of Chemistry Research Center for Analytical Sciences State Key Laboratory of Medicinal Chemical Biology Tianjin Key Laboratory of Molecular Recognition and Biosensing Nankai University Tianjin China; ^5^ Shandong Provincial Key Laboratory of Precision Oncology, Shandong Cancer Hospital and Institute Shandong First Medical University and Shandong Academy of Medical Sciences Jinan China

**Keywords:** contrast agents, CT imaging, Hafnium‐rich carbon dots, preclinical evaluation, spectral CT

## Abstract

Hafnium (Hf) has emerged as a promising element for next‐generation computed tomography (CT) contrast agents owing to its high X‐ray attenuation coefficient and favorable biocompatibility. However, scalable synthesis of Hf‐based imaging probes with sufficiently high metal content for large‐animal preclinical studies remains challenging. Here, we report a facile air‐assisted pyrolysis strategy for the gram‐scale synthesis of Hf‐rich carbon dots (Hf‐rCDs). The resulting Hf‐rCDs exhibit a high Hf content (40.7%), robust batch productivity (>2 g per batch), ultra‐small hydrodynamic size (∼3.2 nm), excellent aqueous solubility (up to 600 mg mL^−^^1^; 244 mg Hf mL^−^^1^), and superior X‐ray attenuation performance. Importantly, Hf‐rCDs demonstrate rapid renal clearance within 2 h post‐injection, supporting a favorable biosafety profile. In vivo CT imaging reveals outstanding contrast enhancement across multiple physiological systems, including the circulatory, urinary, gastrointestinal, and lymphatic systems. Notably, Hf‐rCDs enable high‐resolution visualization of cervical vasculature in swine, highlighting their strong translational potential. Collectively, these results establish Hf‐rCDs as a compelling alternative to conventional iodine‐based contrast agents for advanced CT imaging.

## Graphical abstract

Hafnium (Hf)‐rich carbon dots (Hf‐rCDs) with high metal content (40.7%) and robust batch production capacity (>2 g per batch) are synthesized via a facile air‐assisted pyrolysis method. In vivo CT imaging demonstrates outstanding performance of Hf‐rCDs across multiple physiological systems and enables high‐resolution visualization of swine cervical vasculature, underscoring their strong potential for clinical translation.

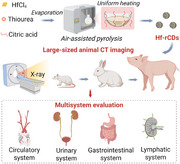

## 1 Introduction

X‐ray computed tomography (CT) is one of the most powerful and widely used imaging techniques in modern clinical practice, characterized by noninvasiveness, high spatial resolution, rapid acquisition, and 3D reconstruction [1, 2]. To enhance the density contrast of interested areas, imaging probes containing high atomic number elements are commonly used for contrast‐enhanced CT imaging [3, 4]. Due to the relatively low sensitivity of CT imaging compared to other imaging modalities, an ideal CT contrast agent should meet several stringent requirements, including simple and high‐yield synthesis, high radiopaque element content, good solubility, rapid clearance, and favorable biocompatibility [5]. Currently, iodine‐based compounds are the most widely used intravenous contrast agents due to the relatively high atomic number (Z = 53) of iodine, high solubility, and good biocompatibility [6]. However, after decades of clinical application, iodine‐based contrast agents have increasingly raised serious concerns due to inadequate sensitivity, renal toxicity, iodine allergies, and contraindications [7, 8].

Since the X‐ray attenuation capabilities of contrast agents are highly dependent on the atomic number of their constituent elements, numerous metallic probes based on high atomic number (Z) elements (e.g., Hf [9], Ta [10, 11, 12, 13], Bi [14, 15, 16, 17], Au [18, 19, 20, 21], Gd [22, 23, 24], Ag [25, 26], Yb [27, 28, 29], Ce [30, 31], Ba [32, 33, 34], and others [35, 36, 37, 38, 39, 40, 41]) have been widely studied over the past few decades, showing promise as alternatives to iodine‐based imaging agents. These high‐Z metallic elements possess stronger X‐ray attenuation coefficients than iodine element, endow high‐Z metallic CT agents with higher sensitivity than clinical iodine‐based CT contrast agents, while also avoiding iodine‐related hypersensitivity. However, current high‐Z metallic CT agents still face two major challenges. The first is their long‐term retention in the body, as probes that cannot be cleared by the kidneys often lead to retention in the liver and spleen, raising safety concerns. The second challenge lies in balancing key physicochemical properties, such as metal content, yield, and solubility. Therefore, developing high‐Z metallic CT agents with excellent overall performance and good biosafety is of great significance and promise, though substantial challenges remain.

Among High‐Z metallic CT agents, Hafnium (Hf, Z = 72)‐based CT imaging probes stand out due to the superior contrast performance, high K‐edge value (Hf 65.4 keV vs. iodine 33.2 keV), and good biocompatibility [42, 43]. Recently, Hf‐based biomaterials have been greatly developed in X‐ray‐related fields, and HfO_2_‐based nanoparticles (NBTXR3) have been approved in the European market as radio‐sensitizing agents for therapy of soft tissue sarcoma [9]. The successful clinical translation and application of NBTXR3 in radiotherapy strongly support the translational potential of Hf‐based probes for CT imaging.

Currently, Hf‐based CT contrast agents are primarily classified into small‐molecule complexes and nanoparticles. Small‐molecule complexes feature defined structures and efficient renal clearance [44, 45] but require complex synthesis that limits clinical translation. Hf‐based nanoprobes, known for their tunable properties and multifunctionality, are typically administered orally or locally for gastrointestinal, bone, joint, lymph node and tumor imaging [46, 47, 48, 49, 50, 51] (Table S1). A subset is given intravenously [52, 53, 54, 55], but they often accumulate in the liver and spleen, leading to prolonged retention and safety concerns. To address these challenges and advance clinical translation, recent studies have focused on developing renal‐clearable Hf‐based nanoprobes. Notably, Su et al. and Liu et al. reported such probes for tumor imaging and radiation protection, respectively [53, 54]. Nevertheless, these systems still face challenges such as low production yields or insufficient metal loading, which limit their suitability for preclinical studies in large animals (Table S1). Therefore, there is an urgent need to develop simple and efficient strategies for producing renal‐clearable Hf‐based nanoprobes with high yields and Hf content to meet translational requirements.

Herein, we report the gram‐scale synthesis of Hf‐rich carbon dots (Hf‐rCDs) via a facile air‐assisted pyrolysis method for CT imaging in medium‐ and large‐sized animals (Figure 1). High‐yield Hf‐rCDs can be readily obtained by heating commercially available precursors in air for 15 min, achieving a batch yield of up to 2 g. The as‐prepared Hf‐rCDs show high metal content (40.7%), ultra‐small hydrodynamic diameter (∼3.2 nm), excellent water solubility (600 mg/mL), and superior X‐ray attenuation capacity. Moreover, Hf‐rCDs demonstrate efficient renal clearance with the majority being excreted within 2 h post‐injection, guaranteeing a favorable biosafety profile. In vivo CT imaging further confirms their excellent performance across multiple physiological systems, such as the circulatory, urinary, gastrointestinal, and lymphatic systems. Notably, preclinical imaging in swine models reveals high‐resolution visualization of cervical vessels, highlighting the strong translational potential of Hf‐rCDs. Collectively, these findings establish Hf‐rCDs as a promising alternative to conventional iodine‐based contrast agents, paving the way for their future application in advanced CT imaging.

## 2 Results

### 2.1 Synthesis and Characterization of Hf‐rCDs

Carbon dots (CDs), a class of carbonaceous nanomaterials with ultra‐small particle size, good solubility, high stability, and favorable biocompatibility, are widely applicable in the biomedical field [56, 57]. The synthesis methods for carbon dots primarily include solvothermal and solid‐phase synthesis. Although solvothermal synthesis is widely used, large‐scale production of carbon dots is limited by the reaction volume and the solubility of the precursors. In solid‐phase synthesis, oil bath heating relies on heat transfer from the bottom of the container, resulting in only the reactants adjacent to the container bottom receiving sufficient heat, making it difficult to achieve uniform heating. In this study, we utilized an air‐assisted pyrolysis strategy to synthesize Hf‐rCDs, in which the reactants were rotary‐evaporated to a semi‐solid state before heating and then placed in an oven for the reaction. The heat is transferred through air, allowing for more uniform heating of the reaction system and ensuring that the precursor materials are evenly heated throughout the process. Meanwhile, no combustion occurred at any stage throughout the entire heating process. The feed ratio of precursors was optimized, and the results showed that Hf‐rCDs synthesized with thiourea:citric acid:hafnium chloride ratio of 9:3:1 exhibited the highest yield (Figure S1).

The ^1^H NMR and diffusion ordered spectroscopy (DOSY) analyses were performed to evaluate purity of Hf‐rCDs. Both Hf‐rCDs and CDs showed broad signals in their ^1^H NMR spectra, and distinct “clouds” of signal intensity in their DOSY spectra (Figure S2), consistent with the inherent inhomogeneity of carbon dots [58, 59, 60]. Besides, a few sharp peaks were observed in the spectra, particularly in the spectrum of CDs, suggesting that trace impurities may remain after purification [61, 62, 63]. From an overall perspective, multiple rounds of dialysis can effectively remove most impurities from the reaction system and enable the large‐scale production of Hf‐rCDs for in vivo CT imaging applications.

The transmission electron microscopy (TEM) image revealed that Hf‐rCDs exhibited high monodispersity and an ultra‐small size, with a hydrodynamic size of approximately 3.2 nm, which was smaller than the renal filtration size (6–8 nm) (Figure 2A; Figure S3A,B). High‐angle annular dark‐field scanning TEM (HAADF–STEM) imaging and the energy‐dispersive spectroscopy (EDS) mapping images obtained by the HAADF–STEM confirmed the homogeneous distributions of C, Hf, N, O, and S elements on the Hf‐rCDs (Figure 2B; Figure S3C). Repeated experiments demonstrated that the laboratory synthesis of Hf‐rCDs can achieve gram‐scale production, with an average single‐batch yield of about 2.6 g, highlighting their potential for large‐scale production (Figure 2C,D). Fourier transform infrared (FT‐IR) spectroscopy revealed a prominent absorption band at 1743 cm^−1^ in both citric acid and undoped carbon dots (CDs), which was attributed to the C═O stretching vibration of carboxylic acid groups. In contrast, this characteristic peak disappeared in the Hf‐rCDs, accompanied by the emergence of a new band at 1633 cm^−1^, which may be attributed to coordination interactions between Hf^4+^ and COO^−^ (Figure 2E) [64]. X‐ray photoelectron spectroscopy (XPS) revealed the presence of C, O, N, and Hf in Hf‐rCDs (Figure 2F). The C 1s spectrum of Hf‐rCDs can be fitted into three peaks at 284.8, 286.4, and 288.4 eV, assigned to C─C/C═C, C─N/C─O, and C═N/C═O, respectively [53, 65, 66, 67] (Figure 2G). The high‐resolution spectrum of N 1s was deconvoluted into three peaks at 399.7, 400.6, and 401.7 eV, which are respectively assigned to pyridinic N, pyrrolic N, and graphitic N (Figure 2H) [65, 66, 67]. Two distinct characteristic peaks for Hf 4f were observed at 17.5 eV (Hf 4f_7/2_) and 19.15 eV (Hf 4f_5/2_), corresponding to the binding energies of Hf^4+^, with a well‐defined separation of 1.7 eV [54] (Figure 2I). Quantitative XPS analysis showed that the atomic percentages of Hf, C, O, and N were 3.16%, 53.46%, 35.01%, and 8.37%, respectively. When converted to mass percentages, the Hf content was 29.95%, slightly lower than the Hf content determined by inductively coupled plasma optical emission spectrometry (ICP‐OES) (40.7%). This discrepancy arises because XPS is a semi‐quantitative technique for surface element analysis.

Synchrotron radiation‐based Hf L3‐edge X‐ray absorption fine structure (XAFS) was further carried out to study the coordination environment of the Hf elements in Hf‐rCDs at the atomic level with commercial HfO_2_ as metal references. Both Hf‐rCDs and HfO_2_ showed same position of absorption edges, which demonstrated the Hf element was +4 valence state in Hf‐rCDs (Figure 2J). In *k* ^2^‐weighted extended X‐ray absorption fine structure (EXAFS) in K‐space and R space (Figure 2K; Figure S4A), both Hf‐rCDs and HfO_2_ showed similar spectra with one main peak at about 1.7 Å, which belonged to Hf─O bonds. Meanwhile, an adjacent peak at 3.1 Å, corresponding to the Hf─O─Hf bonds of HfO_2_, also appeared in the spectrum of Hf‐rCDs. The wavelet transform (WT) analysis also showed the Hf─O bonds and Hf─O─Hf bonds in Hf‐rCDs and HfO_2_ (Figure S4B). In commercial HfO_2_, fitting curves manifested the coordination numbers and Debye–Waller factors of Hf─O bonds were six and 0.0047 Å^2^, respectively (Figure S5A). In Hf‐rCDs, the coordination numbers of Hf─O bonds were 6.07 ± 0.1, and the Debye–Waller factors were 0.0081 Å^2^, which is higher than that of HfO_2_ (Figure S5B). These results demonstrated that the Hf element primarily coordinates with O, with the presence of Hf─O─Hf bonds. Additionally, the distorted coordination numbers and relatively high Debye–Waller factors suggested that the arrangement of Hf─O bonds was relatively disordered.

Optical characterization revealed a distinct absorption peak at 333 nm and excitation‐dependent photoluminescence spanning 350–650 nm. These features were attributed to the surface functional groups and surface states of the Hf‐rCDs, consistent with the typical behavior of carbon dots [68] (Figure S6A,B). X‐ray diffraction (XRD) analysis revealed a diffraction peak at around 23° with a broad background, indicating the weak crystallinity of Hf‐rCDs (Figure 2L). Additionally, the solubility of Hf‐rCDs in aqueous solutions was as high as 600 mg/mL (244 mg Hf/mL), which was sufficient to meet the high concentration requirements for CT imaging probes (Figure 2M). Furthermore, Hf‐rCDs remained well dispersed in pure water, normal saline (NS), phosphate‐buffered saline (PBS, pH 7.4, 10 mM), and cell medium (CM) for at least 14 days without obvious precipitation (Figure S7A). Meanwhile, the leakage of Hf^4+^ in various dispersion media remained below 3% over 14 days (Figure S7B), demonstrating the good colloidal stability and low Hf leakage of the Hf‐rCDs. The above results proved that Hf‐rCDs exhibited large‐scale production capability, good colloidal stability, and remarkably high metal content, making them highly suitable for CT imaging applications.

### 2.2 In Vitro CT Imaging

Current clinical CT agents are predominantly iodine‐based small molecules, which suffer from low sensitivity, poor performance at high monochromatic energy range in spectral CT imaging, and iodine‐associated contraindications. The X‐ray attenuation efficiency of contrast agents strongly depends on the atomic number and K‐edge energy of their constituent elements. Hf has a higher atomic number (Z = 72) and a higher K‐edge energy (65.4 keV) than iodine (Z = 53, K‐edge 33.2 keV), which endows Hf‐rCDs with high sensitivity and robust spectral CT performance, while simultaneously avoiding iodine‐associated contraindications. Solutions of Hf‐rCDs and iohexol were scanned at equivalent concentrations of radiopaque elements (Hf or I), and the CT values of both agents showed a linear correlation with their concentrations (Figure 3A,B; Figure S8). At the same concentration, Hf‐rCDs exhibited comparable imaging performance to iohexol at a tube voltage of 80 kV, but significantly outperformed iohexol at 100, 120, and 140 kV. Under the clinically common tube voltage of 120 kV, Hf‐rCDs exhibited a CT value of 535.82 HU, which was 1.4 times higher than that of iohexol (380.48 HU) at an equivalent concentration of radiopaque elements (100 mm Hf/I) (Figure 3B).

### 2.3 Biosafety Evaluation and in vivo CT Imaging of Rats

Before further proceeding to in vivo CT imaging, the blood half‐life of Hf‐rCDs was determined to be 11.50 min in rats (Figure 3C), which was comparable to that of clinical iodine contrast agents (19.5 min) (Figure S9). Subsequently, the CT imaging was conducted on normal SD rats, and the imaging performance was analyzed in detail. After injection, the kidneys were clearly highlighted and distinctly visible from the surrounding tissues in the images (Figure 3D). At 3 min, remarkable enhancement was also observed in the ureters, and then the enhancement in the kidneys gradually declined, while strong enhancement became evident in the bladder at 10 min. The CT value analysis further confirmed that Hf‐rCDs induced prominent renal enhancement, peaking at 3 min post‐injection and gradually declining thereafter, returning to baseline levels by 60 min (Figure 3E). Meanwhile, the liver exhibited a transient enhancement due to vascular perfusion of Hf‐rCDs without any evidence of accumulation or retention (Figure 3D,E). At about 10 min post‐injection, the bladder showed pronounced enhancement, and the urine appeared deep black, indicating that the Hf‐rCDs flowed into the bladder (Figure 3F).

ICP‐OES biodistribution study was fruther carried out and the results showed that, at 10 min after injection, Hf‐rCDs were primarily concentrated in the kidneys, with some distribution also observed in other organs (Figure S10A). Over time, the concentration of Hf gradually decreased. Meanwhile, a very high concentration of Hf was detected in the urine at 10 min post‐injection and then gradually decreased (Figure S10B). At all time points, no significant Hf signal was detected in the feces. These findings were consistent with the CT imaging results of Hf‐rCDs and demonstrated that Hf‐rCDs were rapidly cleared through the kidneys following intravenous injection. Both CT imaging and ICP‐OES biodistribution analysis confirmed that Hf‐rCDs were renal‐clearable (Figure 3G), ensuring their efficient elimination from the body and minimizing potential safety risks.

To assess the biosafety of Hf‐rCDs, a hemolysis assay was conducted by incubating red blood cells with Hf‐rCDs for 3 h. Even at a high concentration of 500 mg/L, no noticeable hemolysis was observed, and the hemolysis rate in all Hf‐rCDs‐treated groups remained below 5% (Figure S11). Further in vivo evaluation indicated the rats exhibited no abnormalities in eating, drinking, neurological status, and body weight gain during the experimental period (Figure S12A). The biochemical markers, including liver function indicators (AST, ALB, TP, and ALT) and kidney function indicators (CREA and UREA), showed no significant fluctuations compared to the control group (Figure S12B). Meanwhile, there was no significant increase in immune and inflammatory indicators compared with the control group (WBC, Lymph, Mon, Gran, and IgG) after treatment of Hf‐rCDs (Figure S12C,D). Furthermore, histopathological examinations of major organs in rats treated with Hf‐rCDs revealed no apparent inflammation, hemorrhage, lesions, or necrosis (Figure S12E). These results confirmed the good biosafety of Hf‐rCDs.

### 2.4 in vivo CT Imaging of Rats with Unilateral Ureteral Obstruction (UUO)

The imaging capability of Hf‐rCDs for kidney diseases was evaluated in a UUO model (Figure 3H), in which the left ureter was ligated irreversibly, but the right ureter was kept intact. The Hf‐rCDs‐based CT imaging was conducted after ureteral obstruction for 24 h. Prior to Hf‐rCDs injection, it was difficult to distinguish the injured kidney from the CT images. Following Hf‐rCDs administration, the right (normal) kidney exhibited significantly enhanced CT signals, allowing for distinct visualization of the renal pelvis and ureter as early as 3 min post‐injection (Figure 3I–K). By 2 h, most of the Hf‐rCDs had been excreted by the right kidney, and the CT values of the right kidney had nearly returned to baseline levels. In contrast, the left (injured) kidney exhibited a slow and mild enhancement pattern, with CT values progressively increasing over time and showing no signs of decline within the 2 h observation period. Moreover, no significant enhancement was observed in the renal pelvis or ureter of the left kidney, indicating slowed Hf‐rCDs perfusion and impaired renal excretion. Histopathological analysis confirmed significant tissue injury in the left kidney, characterized by marked dilation of renal tubules in both the cortex and medulla, along with localized infiltration of inflammatory cells (Figure 3L). These results demonstrate that Hf‐rCDs exhibit excellent performance in CT imaging of UUO and can serve as imaging probes for the accurate diagnosis of kidney diseases.

### 2.5 in vivo CT Imaging of Medium‐Sized Animals (Rabbits)

Due to their high production yield and excellent CT imaging capabilities, Hf‐rCDs were further subjected to preclinical evaluation in medium‐sized animals (rabbits). After injection of Hf‐rCDs, the dynamic changes in blood Hf element concentration were monitored, and the blood half‐life was calculated to be 32.94 min in rabbits (Figure S13A), which is comparable to that of clinical iodine‐based contrast agents (26.84 min) (Figure S13B). The imaging performance of Hf‐rCDs was evaluated through CT angiography (CTA) of the carotid and thoracoabdominal regions in rabbits. The Hf‐rCDs were injected into the rabbit's marginal ear vein via a high‐pressure syringe at a rate of 1 mL/s (450 mg Hf‐rCDs/kg) and a 4 s delay before scanning. CT imaging demonstrated that Hf‐rCDs produced significant contrast enhancement, enabling clear visualization of the heart chambers, aortic arch, the left and right common carotid arteries (LCCA/RCCA), and left and right subclavian arteries (LSCA/RSCA) (Figure 4A). In contrast, although iohexol‐based CTA was able to delineate the vascular structures at equivalent molar concentrations of radiopaque elements, the images exhibited lower spatial resolution, reduced contrast, and poorly defined vessel boundaries (Figure 4A). Moreover, quantitative analysis showed that Hf‐rCDs‐based CTA provided higher CT attenuation value, SNR, and CNR in neck vessels than those achieved with iohexol‐based CTA (CNR: 26 vs. 5; SNR: 69 vs. 25) (Figure 4B,C; Figure S14A).

Thoracoabdominal vasculature imaging in rabbits was performed by injecting Hf‐rCDs at 2 mL/s (450 mg Hf‐rCDs/kg) using a high‐pressure syringe and a 2 s delay before scanning. The injection produced strong contrast enhancement, distinctly outlining the abdominal aorta, celiac artery, superior mesenteric artery, and left renal artery in the 3D reconstructed images (Figure 4D). In comparison, iohexol injection only allowed clear visualization of the abdominal aorta, with limited ability to depict its distal vessels and branches. Moreover, quantitative analysis showed that the Hf‐rCDs‐based CTA provided higher CT attenuation value, SNR, and CNR in thoracoabdominal vessels than iohexol‐based CTA (CNR: 45 vs. 10; SNR: 35 vs. 20) (Figure 4E,F; Figure S14B). These findings emphasized the superior vascular CT imaging capacity of Hf‐rCDs in medium‐sized animals.

Then, the biosafety of Hf‐rCDs in rabbits was evaluated using multiple methods. After the injection of Hf‐rCDs, no significant abnormalities in behavior, appetite, mental status, or weight gain were observed in rabbits within 28 days (Figure S15A). Additionally, key hepatic and renal function markers did not exhibit significant increases compared with the control group (Figure S15B). Immune and inflammatory‐related indicators showed no significant increase, and histopathological examination of major organs also showed no noticeable lesions, necrosis, or inflammation (Figure S15C–E). Comprehensive toxicity evaluation confirmed the good biocompatibility of Hf‐rCDs in rabbits.

### 2.6 in vivo CT Imaging of Large‐Sized Animals (Swine)

CT imaging studies were conducted on a large mammalian animal (swine) to further assess the potential of Hf‐rCDs for clinical translation (Figure 5A). The Hf‐rCDs solution was administered into the porcine auricular marginal vein using a high‐pressure injector at a rate of 2.5 mL/s (450 mg Hf‐rCDs/kg), followed by 20 mL saline flush at the same flow rate, and a 16 s delay before scanning. Hf‐rCDs circulated to the heart and were pumped through the aorta to enhance arteries at various levels, including subclavian arteries, carotid arteries, and the branches. CT imaging revealed strong enhancement of the heart chambers and major blood vessels in grayscale images, while VR images demonstrated clear delineation of the heart, aortic arch, bilateral carotid arteries, and subclavian arteries following Hf‐rCDs administration (Figure 5B). Compared to Hf‐rCDs‐based CTA, iohexol‐based CTA at equivalent radiopaque element concentrations (Hf or I) resulted in discontinuous arterial visualization and reduced vascular enhancement (Figure 5C).

Curved planar reconstructions of the right/left subclavian arteries (RSCA/LSCA) and right/left common carotid arteries (RCCA/LCCA) further confirmed the superior ability of Hf‐rCDs to depict full‐length vascular structures across multiple anatomical planes (Figure 5D). Quantitative assessments showed significantly higher CT value, CNR, and SNR for Hf‐rCDs compared to iohexol in cervical vessel imaging (Figure 5E; Figure S16A). Meanwhile, the body weight of swine did not show an obvious decrease, and biochemical parameters were dynamically monitored during the imaging process, and the results showed no abnormal elevation in liver or kidney function markers (Figure 5F; Figure S16B). These results demonstrated that Hf‐rCDs enabled high‐quality CTA in large mammalian models, offering detailed anatomical visualization of cervical vessels with excellent safety.

Overall, preclinical evaluations in rabbits and swine demonstrated that Hf‐rCDs are promising and effective CT contrast agents with exceptional imaging performance. Moreover, Hf‐rCDs exhibited superior biosafety profiles across rats, rabbits, and swine, supporting their strong potential for future clinical translation and applications.

### 2.7 Reduction of Metal Artifacts Via Spectral CT Imaging

With the growing use of metallic implants in clinical practice, metal‐induced artifacts on CT scans can severely impair image quality and hinder diagnosis. Spectral CT uses high and low tube voltages to enable monochromatic energy discrimination, effectively reducing metal artifacts in CT imaging by utilizing high‐energy virtual monochromatic imaging, which minimizes beam hardening. However, the X‐ray attenuation of clinical iodine‐based contrast agents decreases significantly at high monochromatic energies, leading to diminished vascular signals in iodine‐enhanced vessels (Figure 6A). Hafnium possesses a relatively high atomic number and K‐edge (Hf: Z = 72, K‐edge = 65.4 keV; I: Z = 53, and K‐edge = 33.2 keV), which shows great potential in minimizing metal artifacts. In vitro spectral CT scans were performed at equivalent concentrations of radiopaque elements (Hf or I), followed by post‐processing to obtain monochromatic energy images. As monochromatic energy increased, CT values of both contrast agents declined, with iohexol showing a more pronounced decline (Figure 6A). In spectral CT imaging of 160 keV, the CT value of Hf‐rCDs was 328.82 HU (100 mM Hf), approximately 12.2 times higher than that of iohexol (26.88 HU, 100 mm I). Then, Hf‐rCDs and iohexol solutions were enclosed in silicone tubes, respectively, and embedded within pork tissue, with metal implants positioned on the tissue surface, followed by conventional CT (tube voltages 70–140 kV) and spectral CT scanning. In conventional CT scans, metal implants generated prominent artifacts across all tube voltages, severely obscuring the visualization of both Hf‐rCDs and iohexol. However, spectral CT images acquired at high monochromatic energies effectively eliminated metal artifact interference. At these energies, iohexol exhibited very low CT values, making enhancement difficult to detect, while Hf‐rCDs maintained clear and distinct enhancement (Figure 6B).

Metal implants are commonly used for fixation and repair in spinal surgeries, so a paraspinal metallic implant model was employed to assess the performance of Hf‐rCDs‐based spectral CT. The model was established by placing a metal implant parallel to the right side of spine and embedding it in the back muscles of rats. Spectral CT imaging without contrast agents revealed that metal implant artifacts obscured the adjacent right kidney at a low monochromatic energy of 40 keV, but these artifacts were markedly reduced at higher energies (80–160 keV) (Figure 6C). In contrast‐enhanced spectral CT with Hf‐rCDs, the kidneys exhibited strong and sustained enhancement across the full energy range (40–160 keV), allowing for clear, artifact‐free renal imaging. However, when using iohexol, the enhancement significantly diminished at higher energies (80–160 keV), compromising the quality of artifact‐free imaging.

Additionally, the imaging performance of Hf‐rCDs in a rabbit model of cervical vasculature with metal implants was evaluated. A metal pin was placed transversely on the rabbits' neck, and Hf‐rCDs were injected at 1 mL/s (450 mg Hf‐rCDs/kg) through the ear marginal vein with a 4 s delay before scanning. At low monochromatic energies (40 and 80 keV), both Hf‐rCDs and iohexol groups showed obvious enhancement in the carotid arteries. However, severe metal artifacts significantly interfered with the visualization of vascular structures and trajectories. As the monochromatic energy increased, metal artifacts were markedly suppressed in high‐energy images. However, in the iohexol group, arterial enhancement declined markedly at energies above 120 keV. In contrast, the Hf‐rCDs group maintained robust carotid artery enhancement even at high monochromatic energies, enabling clear, artifact‐free vascular imaging (Figure 6D).

Two quantitative parameters were calculated based on signal measurements at three locations. S_1_: the artery signal adjacent to the metal implant; S_2_: the artery signal unaffected by the metal implant; S_3_: the muscle signal adjacent to the metal implant. Theoretically, the closer S_1_/S_2_ value is to 1.0, the less severe the artifacts, while the higher S_1_/S_3_ value represents the better vascular contrast. The results showed that S_1_/S_2_ for both Hf‐rCDs group and iohexol group followed a similar trend, approaching 1.0 as the monoenergetic level increased, indicating progressively reduced artifact interference at high‐monoenergetic images (Figure 6E). S_1_/S_3_, which reflects the contrast between the vessel and surrounding tissues. Compared with the iohexol group, Hf‐rCDs showed higher S_1_/S_3_ values at 120 and 160 keV, demonstrating that the vessels of Hf‐rCDs group maintained good contrast at high monoenergies (Figure 6F). Consequently, vessels in the Hf‐rCDs group sustained stronger enhancement in high‐monoenergetic images, outperforming those in the iohexol group. At 120 kV, artifacts induced by the metallic implants significantly impaired the visualization of the underlying vasculature in both the Hf‐rCDs and Iohexol groups (Figure 6G). These results demonstrated that Hf‐rCDs‐based spectral CT enabled artifact‐free contrast‐enhanced imaging of the kidneys and vasculature, even in the presence of metal implants.

### 2.8 in vivo Gastrointestinal and Lymph Node CT Imaging

Accurate diagnosis of gastrointestinal diseases often relies on the use of CT contrast agents. In addition to intravenous injection for circulatory system and urinary system CT imaging, we investigated the feasibility of Hf‐rCDs for imaging the gastrointestinal and lymphatic systems via oral gavage and subcutaneous injection. Following oral administration of Hf‐rCDs in rats, the gastrointestinal tract exhibited time‐dependent enhancement, with the stomach clearly visible at 5 min post‐gavage, the small intestine at 30 min, and the colon at 2 h. By 24 h, the Hf‐rCDs were nearly fully excreted, and complete clearance from the gastrointestinal tract was observed by day 3 (Figure 7A).

A partial small intestine obstruction model was established to further evaluate the gastrointestinal imaging capabilities of Hf‐rCDs (Figure 7B). The results showed that 30 min after oral gavage, the stenotic region of the small intestine was clearly visualized from both ventral and dorsal views, and the imaging findings were consistent with the obstruction site identified during post‐mortem dissection (Figure 7C). Additionally, histological analysis confirmed that oral administration of Hf‐rCDs caused no observable damage to the stomach, small intestine, or large intestine, supporting their excellent gastrointestinal safety (Figure 7D).

Lymphatic circulation is a vital part of the human body, responsible for fluid balance, immune surveillance, and waste transport. CT imaging capability of Hf‐rCDs for lymph nodes was investigated via subcutaneous injection of Hf‐rCDs (Figure 7E). Before the injection of Hf‐rCDs, the inguinal lymph node could not be accurately visualized in terms of their location and size by CT imaging (Figure 7F). After subcutaneous injection of Hf‐rCDs into the left paw, a high‐intensity nodule was clearly observed in the left inguinal region, and the structure of the nodule was clearly visualized in the VR. H&E staining confirmed that the enhanced nodule was a lymph node, primarily due to the transport of Hf‐rCDs into the lymphatic system (Figure 7G). By 3 h post‐injection, the signal nearly returned to baseline, indicating further transport and metabolism of Hf‐rCDs within the lymphatic system. These findings suggest that Hf‐rCDs hold significant potential for CT imaging of the lymphatic circulation system.

## 3 Conclusion

In this work, we developed a facile air‐assisted pyrolysis method for the gram‐scale synthesis of Hf‐rich carbon dots (Hf‐rCDs) with high metal content for multi‐system CT imaging. The resulting Hf‐rCDs exhibit an ultra‐small hydrodynamic size, high Hf loading, excellent aqueous solubility, strong X‐ray attenuation, and robust scalability. In vivo CT imaging demonstrated outstanding contrast enhancement across the circulatory, urinary, gastrointestinal, and lymphatic systems. Notably, preclinical studies in swine enabled high‐resolution visualization of cervical vasculature, underscoring the translational potential of Hf‐rCDs. Moreover, Hf‐rCDs maintained stable imaging performance across a range of tube voltages, and spectral CT imaging effectively reduced metal artifacts in spectral CTA. Future studies will systematically evaluate the impact of purification strategies on Hf‐rCDs purity, yield, and imaging performance in multisystem disease models in large animals. Overall, this work provides a promising strategy for developing high‐performance, non‐iodinated CT contrast agents and establishes a strong preclinical foundation for clinical translation.

## 4 Experimental Section

### 4.1 Materials

Hafnium tetrachloride was supplied by Fujian Chunming New Material Technology Co., Ltd. (Fujian, China). Thiourea (99%) and anhydrous citric acid (99.5%) were obtained from Shanghai Aladdin Biochemical Technology Co., Ltd. (Shanghai, China). Ultrapure water was sourced from Wahaha Co., Ltd. (Hangzhou, China). All reagents and materials were used as received without further purification.

### 4.2 Synthesis of Hf‐rCDs

Hf‐rCDs were synthesized using an air‐assisted pyrolysis strategy, as an improvement of previously reported method [53]. In a typical procedure, thiourea (1.37 g) and anhydrous citric acid (1.15 g) were added to a 2000 mL single‐neck round‐bottom flask, followed by the addition of a hafnium tetrachloride solution (0.64 g in 100 mL), corresponding to a thiourea:citric acid:hafnium chloride molar ratio of 9:3:1. The mixture was stirred at room temperature for 15 min, followed by rotary evaporation to a semi‐solid state. Subsequently, the flask was heated in an oven at 240 °C for 15 min. After cooling to room temperature, 160 mL of ultrapure water was added to the flask and sonicated with an ultrasonic frequency of 40 kHz and 200 W for 30 min, while manually shaking to ensure the crude products were completely dispersed. Then, the mixture was centrifuged (10619 g for 5 min), and the supernatant was collected for subsequent dialysis purification. The supernatant was evenly divided into eight dialysis bags (1000 Da molecular weight cut off) and dialyzed against ultrapure water in 500 mL beakers, with the water replaced every 4 h for a total of four times. The dialysate was then filtered through 220 nm filter membrane and rotary‐evaporated at 80 °C and 60 rpm until dry. The resulting products were collected and stored at 4 °C for subsequent experiments. For the synthesis of CDs nanoparticles, the HfCl_4_ was not added to the reaction, and other steps were the same as those of Hf‐rCDs synthesis.

### 4.3 In Vitro CT Imaging Performance of Hf‐rCDs

The CT imaging performance of Hf‐rCDs was evaluated by scanning solutions with varying concentrations (0, 25, 50, 75, and 100 mmol Hf/L) using a clinical dual‐energy CT (DECT) system (Somatom Drive, Siemens Healthineers, Erlangen, Germany). Scanning parameters included slice thickness of 0.5 mm, adaptive tube current, and X‐ray tube voltage ranging from 80 to 140 kV. For spectral CT imaging, Hf‐rCDs were scanned with parameters set to slice thickness of 0.5 mm, adaptive tube current, and dual tube voltages of 90/150 Sn. Syngo software (Siemens Healthineers, Germany) was used to process spectral images at monochromatic X‐ray energies ranging from 40 to 180 keV. For comparison, imaging experiments were performed using iohexol at equivalent Hf/I concentrations.

### 4.4 Animal Models

All animal experiments were approved by the Animal Care and Use Committee of Tianjin Medical University General Hospital (IRB2022‐DW‐76) and Nankai University (2024‐SYDWLL‐000794). Male Sprague‐Dawley (SD) rats weighing 200–220 g were purchased from SPF Biotechnology Co., Ltd. Female New Zealand white rabbits weighing 2.0–2.2 kg were purchased from Tianjin Yuda Experimental Animal Breeding Co., Ltd. Swine weighing 20–22 kg were purchased from Tianjin Bainong Laboratory Animal Breeding Technology Co., Ltd.

### 4.5 Determination of Blood Half‐Life

The pharmacokinetics of Hf‐rCDs were investigated in rats (600 mg Hf‐rCDs/kg, *n* = 3) and rabbits (450 mg Hf‐rCDs/kg, *n* = 3) following intravenous injection. Blood samples from rats or rabbits were collected at different time intervals. Each blood sample was centrifuged to separate the plasma and diluted. Hafnium content was quantified using ICP‐OES, and the blood half‐life of Hf‐rCDs was calculated using one‐compartment decay mode.

### 4.6 In Vivo CT Imaging of Normal Rats and UUO Rat Models

Establishment of a unilateral ureteral obstruction (UUO) rat model: male SD rats (200–220 g) were anesthetized with isoflurane, and the left ureter was exposed via a left flank incision, and ligated with nonabsorbable sutures. After suturing the wounds of UUO rats, they were kept under a normal diet, and the CT imaging was carried out 1 day post ligation. Normal or UUO model rats were subjected to CT imaging before and after intravenous injection of Hf‐rCDs (600 mg Hf‐rCDs/kg, *n* = 3). Scanning parameters included slice thickness of 0.5 mm, adaptive tube current, and tube voltage of 120 kV. CT images were captured at various time points (pre‐administration, 30 and 90 s; 3 and 10 min; 1 and 2 h).

### 4.7 In Vivo CT Imaging of Rabbits

The imaging capability of Hf‐rCDs in rabbits was evaluated using both cervical artery CT angiography (CTA) and thoracoabdominal artery CTA. Rabbits were anesthetized with isoflurane and positioned centrally in the scanner to optimize the field of view. A 24G indwelling needle was inserted into the ear vein and connected to a high‐pressure injector (Empower CTA+). Initial non‐contrast CTA scans were performed with the following parameters: slice thickness of 0.5 mm, adaptive tube current, and tube voltage of 120 kV. For carotid artery CTA, scans were initiated 4 s after contrast injection at 1 mL/s (450 mg Hf‐rCDs/kg). For thoracoabdominal CTA, scans were initiated 2 s after the contrast injection at 2 mL/s (450 mg Hf‐rCDs/kg). For comparison, imaging experiments were performed using iohexol at equivalent Hf/I concentrations.

### 4.8 In Vivo CT Imaging of Swine

The imaging capability of Hf‐rCDs in swine was conducted using cervical artery CTA. The swine was anesthetized with isoflurane and positioned centrally in the scanner to optimize the field of view. A 22G indwelling needle was inserted into the ear vein and connected to a high‐pressure injector (Empower CTA+). Initial non‐contrast CTA scans were performed with the following parameters: slice thickness of 0.5 mm, adaptive tube current, and tube voltage of 120 kV. Contrast agents were administered intravenously at 2.5 mL/s (450 mg Hf‐rCDs/kg). For carotid artery CTA, scans were initiated 16 s after contrast injection. For comparison, imaging experiments were performed using iohexol at equivalent Hf/I concentrations.

### 4.9 Reduction of Metal Artifacts Via Spectral CT Imaging

Hf‐rCDs and iohexol solutions were separately encapsulated in silicone tubes, embedded in porcine tissue with superficial metal implants, and subjected to both conventional CT (70–140 kV) and spectral CT scanning. Scans were performed with the following settings: slice thickness of 0.5 mm, adaptive tube current, and tube voltage of 70–140 kV. Spectral CT scan was conducted with 80/150 Sn.

A paraspinal metallic implant model‐clinically relevant for spinal fixation was established by positioning a metal implant (titanium‐niobium intramedullary nail) parallel to the right lateral aspect of the spine within rat dorsal musculature to evaluate Hf‐rCDs‐based spectral CT performance. Conventional CT and spectral CT imaging were performed before and after intravenous injection of Hf‐rCDs (600 mg Hf‐rCDs/kg).

A metallic implant model was established by horizontally positioning a titanium‐niobium alloy intramedullary nail anterior to a rabbit's neck. Rabbits were anesthetized with isoflurane and positioned centrally in the scanner to optimize the field of view. A 24G indwelling needle was inserted into the ear vein and connected to a high‐pressure injector (Empower CTA+). Both the conventional CTA and spectral CTA were initiated 4 s after contrast injection at 1 mL/s (450 mg Hf‐rCDs/kg). Conventional CTA imaging parameters were identical to those described in the previous section, “In Vivo CT Imaging of Rabbits”. Spectral CTA was acquired using the following parameters: 0.5 mm slice thickness and 80/150 Sn.

All spectral CT data were automatically reconstructed into three datasets (80 and 150 kV, and combined 80/150 kV images) with a slice thickness of 0.5 mm on a commercial workstation (Syngo.via; Siemens Healthineers, Germany).

### 4.10 In Vivo Gastrointestinal CT Imaging

Rats were anesthetized with isoflurane and positioned centrally in the CT scanner to optimize the field of view. The rats were gavaged with Hf‐rCDs (5 mL, 3 g Hf‐rCDs/kg, *n* = 3) and scanned before and after administration of Hf‐rCDs at 5 and 30 min; 1 and 2 h; 1 and 3 days. CT imaging was performed with standard scanning parameters: slice thickness of 0.5 mm, adaptive tube current, and tube voltage of 120 kV. After imaging, H&E staining, and histopathological evaluation were performed on the stomach, small intestine, and large intestine.

### 4.11 Construction and Imaging of the Partial Small Intestine Obstruction Model

SD rats were anesthetized with isoflurane and fixed in a supine position. Abdominal hair was removed using a depilatory cream. A midline incision was made on the abdomen, and the muscle and fascia were carefully separated to expose the duodenum. Partial small intestinal obstruction was induced by loosely ligating the intestine with a 3–0 silk suture. Afterward, the abdominal skin was sutured, and the surgical area was thoroughly cleaned. The scanning parameters were the same as those described in the preceding paragraph.

### 4.12 In Vivo Lymph Node CT Imaging

Rats were anesthetized with isoflurane and subcutaneously injected with Hf‐rCDs (500 mg Hf‐rCDs/kg, *n* = 3). CT scanning was carried out before and after the administration of Hf‐rCDs. Scanning parameters: slice thickness of 0.5 mm, adaptive tube current, and tube voltage of 120 kV. After imaging, H&E staining, and histopathological evaluation were performed on the lymph node.

### 4.13 Calculation of SNR and CNR

To quantify imaging quality, the CT values and standard deviations (SD) of background were measured by Bee DICOM VIEWER software. Signal‐to‐noise ratio (SNR) and contrast‐to‐noise ratio (CNR) were calculated as follows: SNR = CT value of ROI / SD of background; CNR = (CT value of ROI–CT value of muscle) / SD of background. ROI represents specific regions of interest.

### 4.14 Statistical Analysis

Statistical analysis data are expressed as mean ± standard deviation (SD) as indicated in the figure captions. Statistical significance was evaluated using Student's t‐test, with significance levels denoted as follows: *p < 0.05, **p < 0.01, ***p < 0.001, and ^****^ *p* < 0.001. All tests were carried out by GraphPad Prism software (v8.0).

## Funding

National Natural Science Foundation of China (82573346, C.Z.), National Natural Science Foundation of China (82272052, J.P.), Tianjin Major Science and Technology Project for Public Health (24ZXGQSY00100, C.Z.), Natural Science Foundation of Tianjin City (25JCYBJC00120, J.P.), Scientific Research Project of Tianjin Education Commission (2023ZD020, S.‐K.S.), Scientific Research Project of Tianjin Education Commission (2024KJ226, Q.Z.)

## Conflicts of Interest

The authors declare no conflicts of interest.

## Supporting information




**Supporting File**: advs73809‐sup‐0001‐SuppMat.docx.

## Data Availability

The data that support the findings of this study are available in the supplementary material of this article.
