# Supplementary material for: Gram‐Scale Synthesis of Hafnium‐Rich Carbon Dots for Preclinical Computed Tomography Imaging Across Various Systems
Source: Adv Sci (Weinh). 2026 Jan 31;13(17):e17986. doi: 10.1002/advs.202517986 (PMC13042817; doi:10.1002/advs.202517986)
Supplement: Supplementary file 1 — Supporting File: advs73809‐sup‐0001‐SuppMat.docx. [file ADVS-13-e17986-s001.docx]

Supporting Information

**Gram-Scale Synthesis of Hafnium-Rich Carbon Dots for Preclinical Computed Tomography Imaging Across Various Systems**

*Shuo Li, Hengrui Wu, Jianqi Deng, Qiyu Sun, Yuping Zhang, Cai Zhang, Jinbin Pan, Dingbin Liu, Xuejun Zhang*, Quan Zou*, Xiaoyuan Chen*, and Shao-Kai Sun**

S. Li, J. Deng, Q. Sun, Y. Zhang, X. Zhang, Q. Zou, S.-K. Sun

School of Medical Imaging, Division of Medical Technology, Tianjin Key Laboratory of Functional Imaging

Tianjin Medical University

Tianjin, China

E-mail: zhangxj@tmu.edu.cn (X. Zhang), zouquan@tmu.edu.cn (Q. Zou), shaokaisun@tmu.edu.cn (S.-K. Sun)

H. Wu, J. Pan

Department of Radiology, Tianjin Key Laboratory of Functional Imaging

Tianjin Medical University General Hospital

Tianjin, China

C. Zhang

Department of Radiology

Tianjin Medical University Cancer Institute and Hospital

National Clinical Research Center of Cancer

Tianjin’s Clinical Research Center for Cancer

Tianjin, China

D. Liu

College of Chemistry, Research Center for Analytical Sciences, State Key Laboratory of Medicinal Chemical Biology, Tianjin Key Laboratory of Molecular Recognition and Biosensing

Nankai University

Tianjin, China

X. Chen

Shandong Provincial Key Laboratory of Precision Oncology, Shandong Cancer Hospital and Institute

Shandong First Medical University and Shandong Academy of Medical Sciences

Jinan, China

E-mail: chen9647@gmail.com (X. Chen)

* Corresponding authors.

**Experimental Section**

*Characterization*: The morphology of Hf-rCDs was characterized by transmission electron microscopy (TEM), with low-magnification image acquired using a HT7700 microscope (Hitachi, Japan) and higher-magnification image obtained using a JEM-2800 microscope (JEOL, Japan). Hydrodynamic diameter was measured using a Zetasizer Nano S90 instrument (Malvern Instruments Ltd., UK). High-angle annular dark-field scanning transmission electron microscopy (HAADF-STEM) was performed on a JEM-ARM300F2 microscope. Fourier-transform infrared (FT-IR) spectra (400-4000 cm^-1^) were recorded on a Nicolet iS10 spectrometer (Madison, USA), using pure KBr as background reference. X-ray diffraction (XRD) pattern was acquired using an Ultima IV diffractometer (Rigaku, Japan). Hafnium content was analyzed by inductively coupled plasma-optical emission spectroscopy (ICP-OES; Agilent 5800 VDV, USA). X-ray photoelectron spectroscopy (XPS) spectra were obtained using an Axis Ultra DLD spectrometer (Kratos Analytical Ltd., UK). Hf L3-edge X-ray absorption fine structure (XAFS) analyses were performed with Si(111) crystal monochromators at the BL14W Beam line at the Shanghai Synchrotron Radiation Facility (SSRF) (Shanghai, China). Hf L3-edge extended X-ray absorption fine structure (EXAFS) spectra were recorded in transmission mode. UV-vis absorption spectra of Hf-rCDs were measured on a UV-3600 plus spectrophotometer (Shimadzu, Japan). Fluorescence spectra were recorded on an F7000 fluorescence spectrophotometer (Hitachi, Japan). ^1^H Nuclear magnetic resonance (NMR) spectra were obtained on a 400 MHz Bruker Avance (III) spectrometer. Diffusion Ordered Spectroscopy (DOSY) measurements were performed on a Bruker 600 MHz NMR spectrometer (Bruker, Germany), with deuterium oxide (D_2_O) employed as the solvent.

*Stability Evaluation of Hf-rCDs*: To assess the colloidal stability, Hf-rCDs were dissolved in ultrapure water, normal saline (NS), phosphate-buffered saline (PBS, pH 7.4, 10 mM), and complete cell medium (CM), and photographed daily for 14 days to monitor stability.

*Hf Leakage Analysis*: Hf-rCDs were dissolved in ultrapure water, normal saline, PBS, and cell medium, respectively (2.5 mg/mL). At different time points (4 h, 1 day, and 14 day), aliquots of each solution were taken and evaporated to dryness. The residues were treated with anhydrous ethanol and vigorously shaken, followed by centrifugation to collect the supernatant, which was then re-evaporated to dryness. Finally, the residues were dissolved in 5% HNO_3_, and the amount of Hf released was determined by ICP-OES.

*Hemolysis Test*: Red blood cells were separated and washed several times with normal saline by centrifugation. Then, Hf-rCDs solutions (0.7 mL) were co-incubated with diluted red blood cells (0.3 mL) at a final concentration of (100, 200, 300, 400, and 500 mg/L) for 3 h at 37 °C. Normal saline (NS) and water of the same volume were used as negative and positive controls, respectively. Subsequently, the mixture was centrifuged at 4500 rpm for 10 min and photographed. The supernatant was then collected and diluted by the same factor, and the absorbance at 541 nm was measured using a 96-well plate reader. The hemolysis rates of Hf-rCDs at various concentrations were calculated by defining the hemolysis induced by the positive and negative controls as 100% and 0%, respectively.

*Biodistribution of Hf-rCDs*: SD rats were intravenously injected with Hf-rCDs (600 mg Hf-rCDs/kg, n = 3) and sacrificed at different time points. Subsequently, major organs (heart, liver, spleen, lung, and kidney) and excreta (urine and feces) were harvested. Each organ or excreta was digested with concentrated nitric acid, and the Hf content was quantified by ICP-OES. Then, the mass of Hf was calculated and Hf-to-tissue (or excreta) weight ratio was determined.

*In Vivo Toxicity Evaluation*: Healthy SD rats were intravenously injected with Hf-rCDs (600 mg Hf-rCDs/kg), and body weight was monitored (n = 3). Blood samples and major organs were collected on day 28 post-injection. Liver function analysis (AST, ALB, TP, and ALT), kidney function analysis (CREA and UREA) was determined with a Mindray BS430 automatic biochemical analyzer. Complete blood count analysis (WBC, Lymph, Mon, Gran) was carried out on a Mindray BC-2800vet hematology analyzer. IgG of rat was determined with Elisa method (lgG (Total) Rat Uncoated ELlSA Kit, Catalog Number 88-50490). Major organs (heart, liver, spleen, kidneys, lungs) were fixed in 4% formaldehyde, sectioned, and stained with hematoxylin and eosin (H&E). In the safety assessment of Hf-rCDs in rabbits, the injection dose was 450 mg Hf-rCDs/kg, with the ear marginal vein used as both the injection and blood collection site. IgG of rabbits was determined with a Rayto Chemray automatic biochemical analyzer. The remaining procedures were identical to those performed in the SD rat evaluation.

**Supplementary Figures**


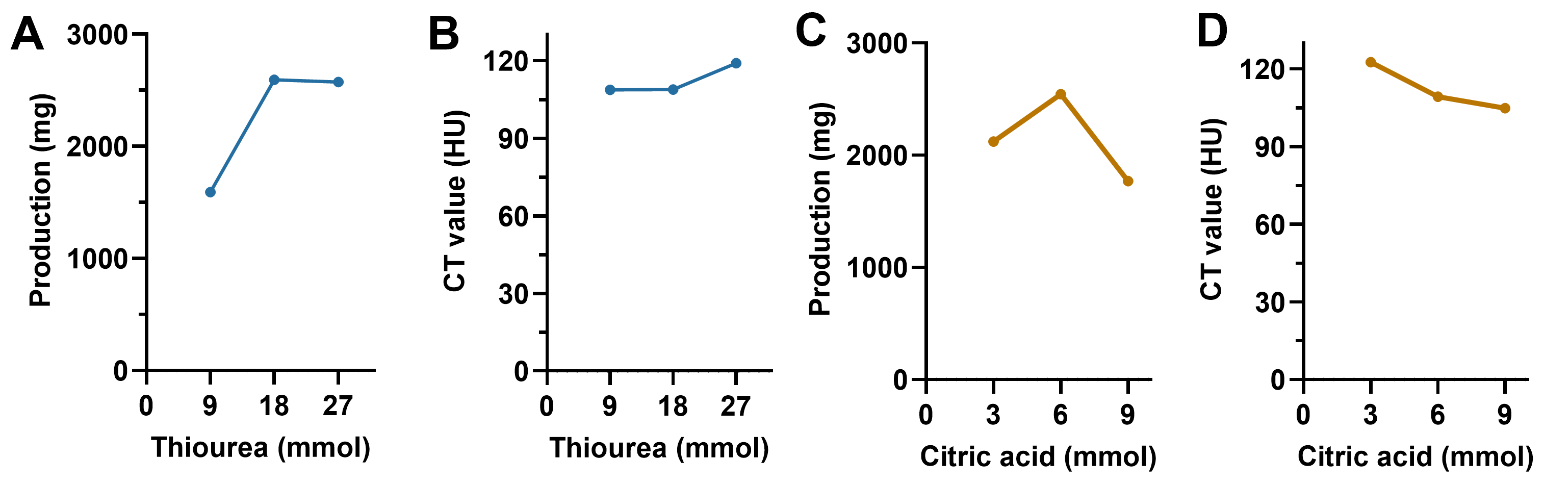


**Figure S1.** Production (A) and CT value (B) of Hf-rCDs synthesized with different amounts of thiourea (citric acid and HfCl_4_ were fixed at 6 mmol and 2 mmol, respectively). Production (C) and CT value (D) of Hf-rCDs synthesized with different amounts of citric acid (thiourea and HfCl_4_ were fixed at 18 mmol and 2 mmol, respectively).


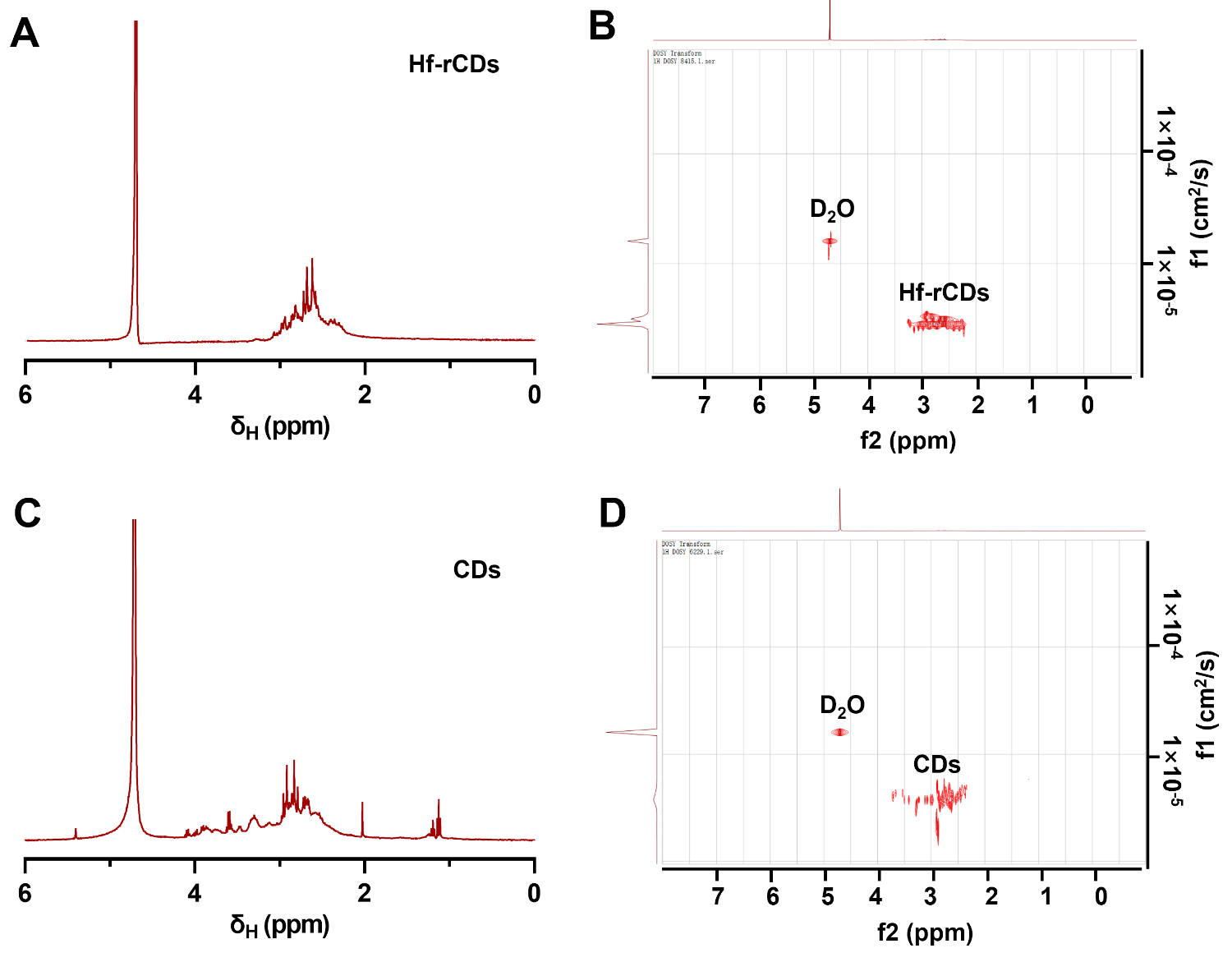


**Figure S2.** ^1^H NMR (D_2_O, 400 MHz) (A) and diffusion ordered spectroscopy (DOSY) spectrum (D_2_O, 600 MHz) (B) of Hf-rCDs. ^1^H NMR (D_2_O, 400 MHz) (C) and DOSY spectrum (D_2_O, 600 MHz) (D) of carbon dots (CDs).

**
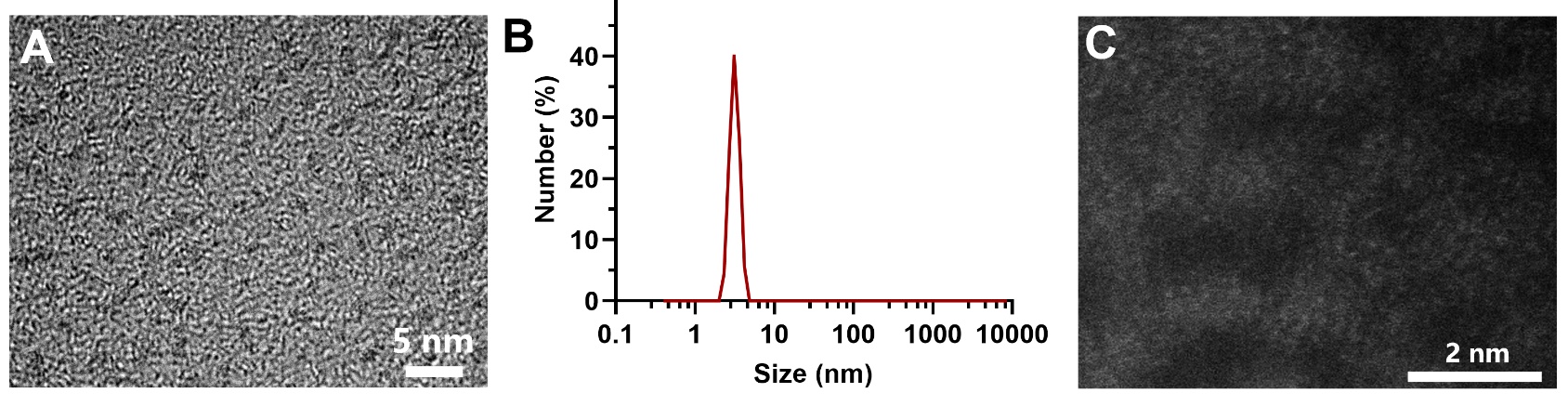
**

**Figure S3.** (A) TEM image of Hf-rCDs. (B) Hydrodynamic size of Hf-rCDs determined by DLS. (C) HAADF-STEM image of Hf-rCDs.


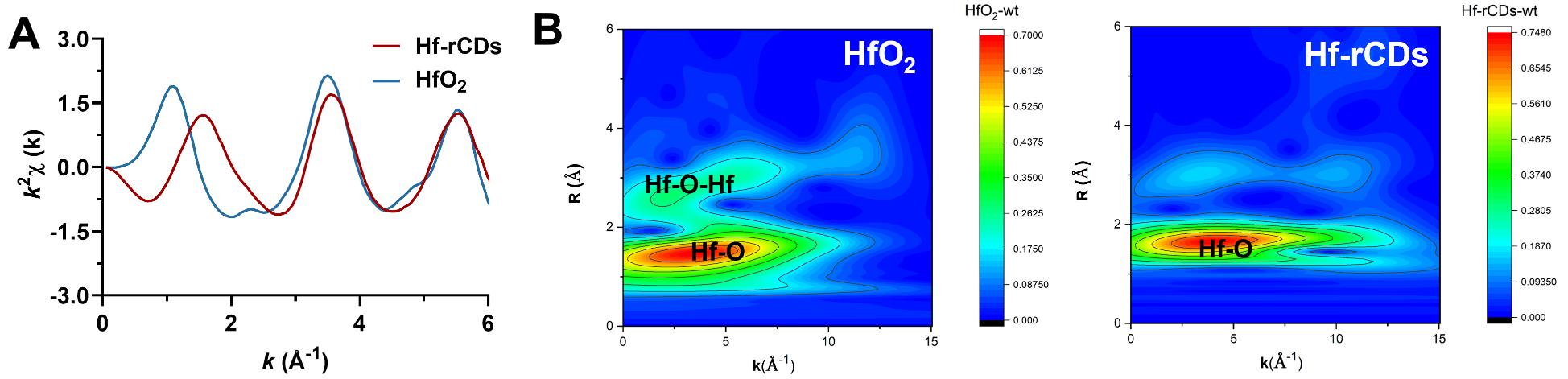


**Figure S4.** (A) The k^2^-weighted EXAFS in K-space of Hf-rCDs and HfO_2_. (B) Wavelet transform (WT) analysis of Hf-rCDs and HfO_2_.


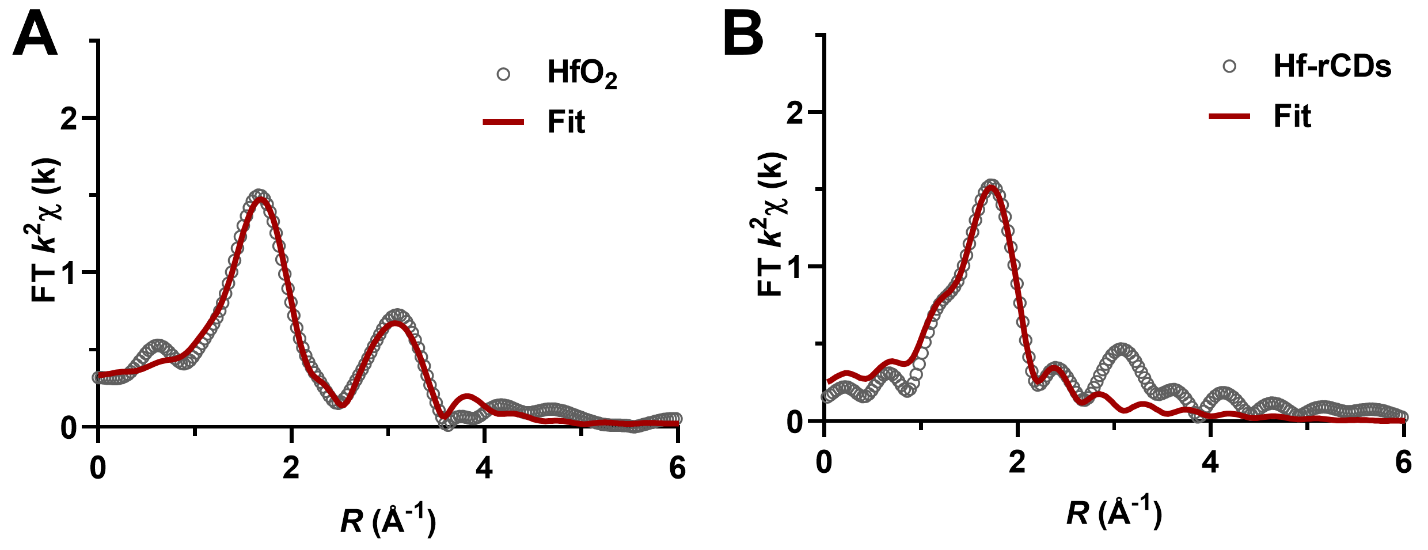


**Figure S5.** Hf R-space fitting curve of HfO_2_ (A) and Hf-rCDs (B) (*Ѕ*_0_^2^ = 0.70).


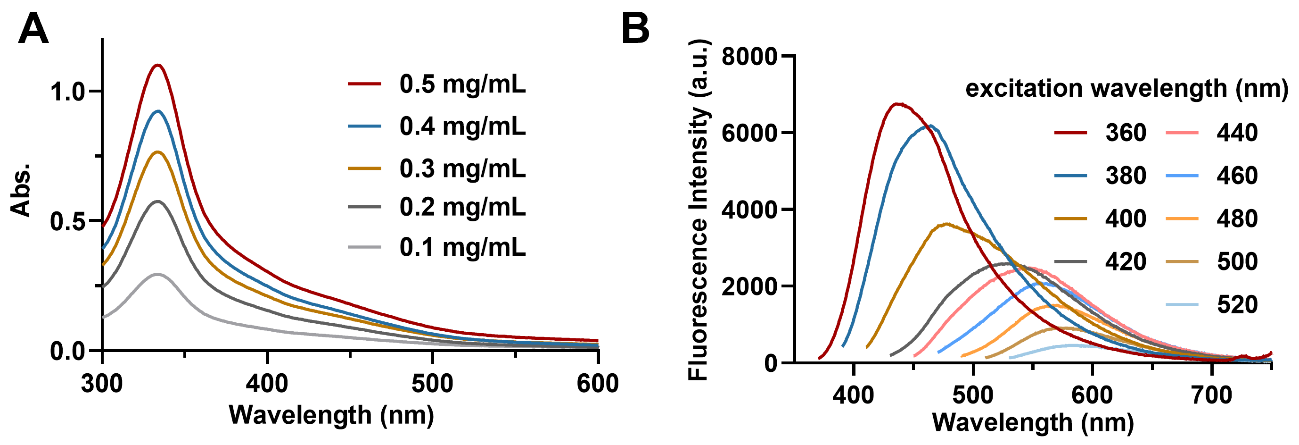


**Figure S6.** (A) Absorption spectra of different concentrations of Hf-rCDs solution. (B) Fluorescence emission spectra of Hf-rCDs under different excitation wavelengths.


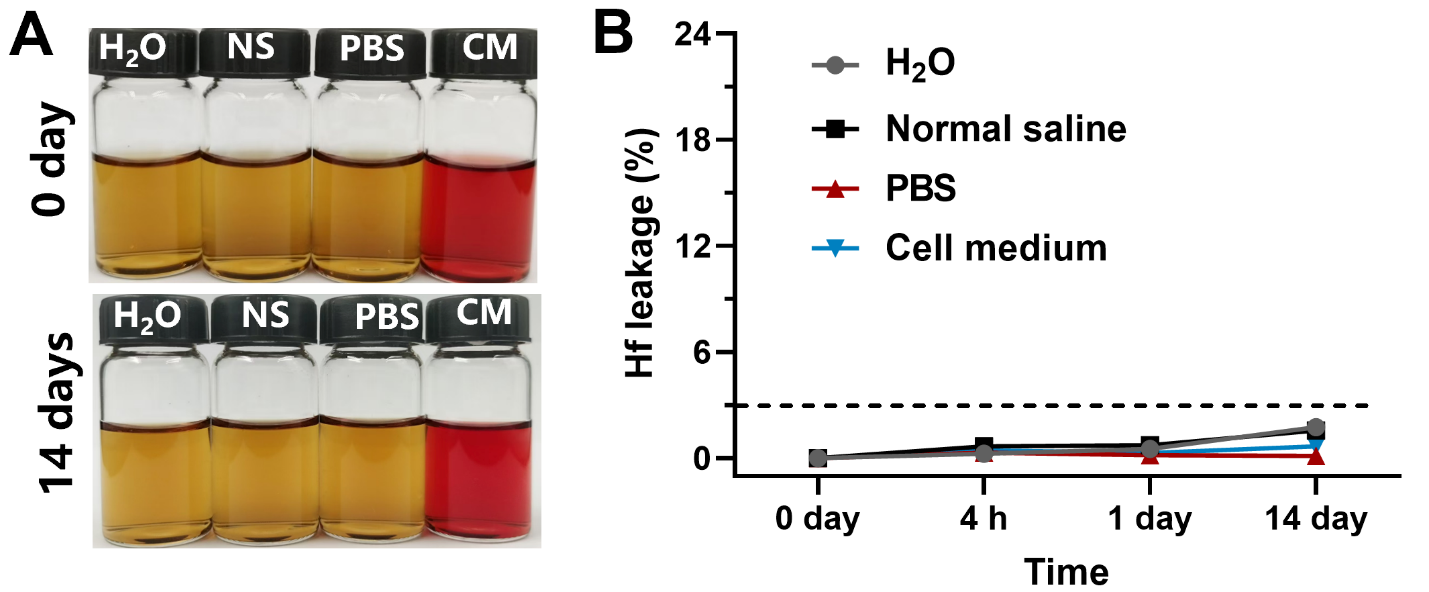


**Figure S7.** (A) Colloidal stability of Hf-rCDs on 0 and 14 days. (B) Measurement of Hf leakage from Hf-rCDs after dispersing in various media for different time periods.

**
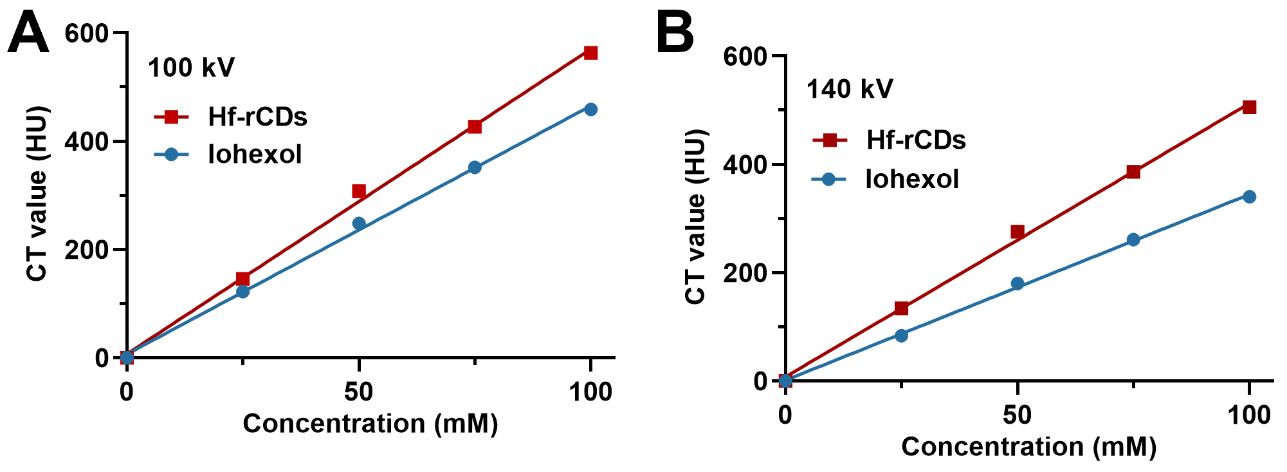
**

**Figure S8.** CT value change curves of Hf-rCDs and iohexol with different concentrations (0-100 Hf or I mmol/L) under tube voltages of 100 kV (A) and 140 kV (B).


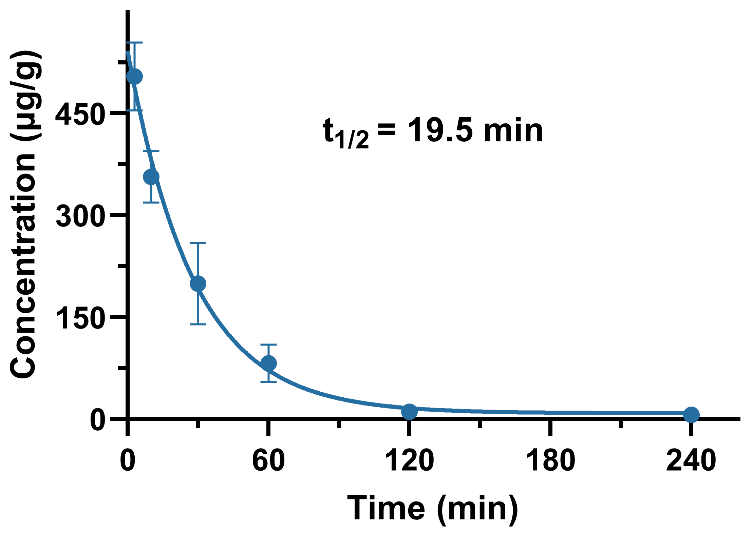


**Figure S9.** Blood half-life of iohexol in rats (n = 3; mean ± SD).


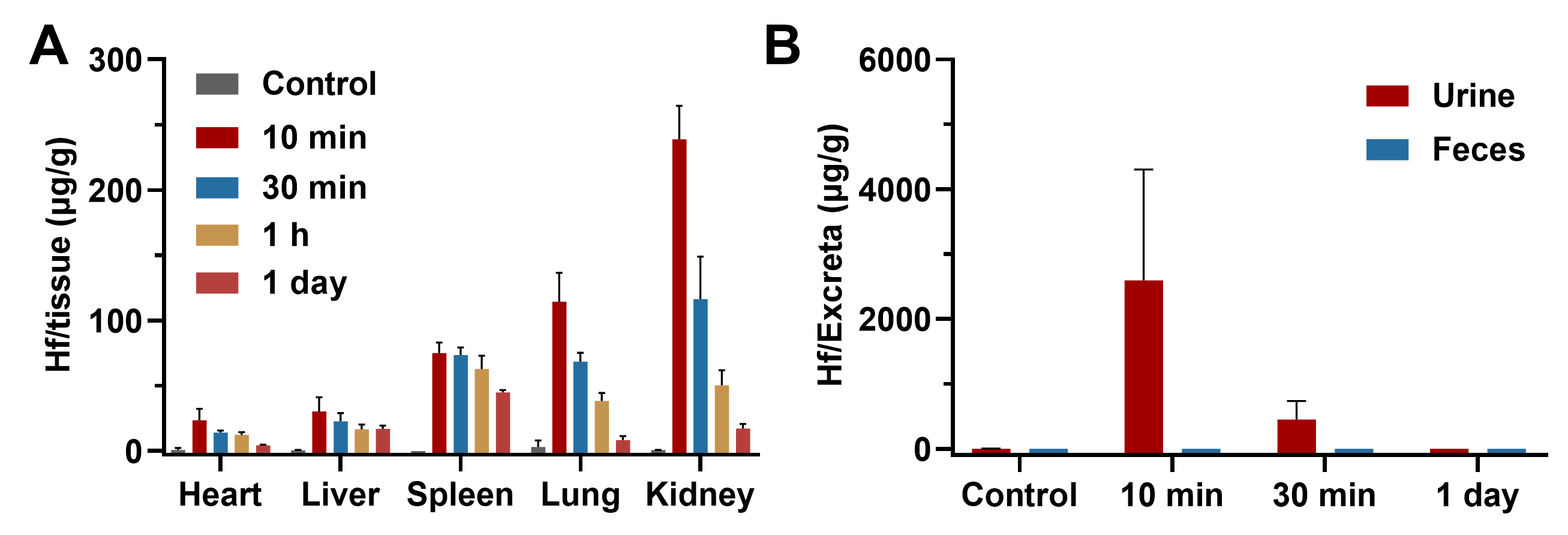


**Figure S10.** Time-dependent quantification of Hf in various organs (A) and excretion (B) of SD rats at different time points post-injection of Hf-rCDs (n = 3; mean ± SD).


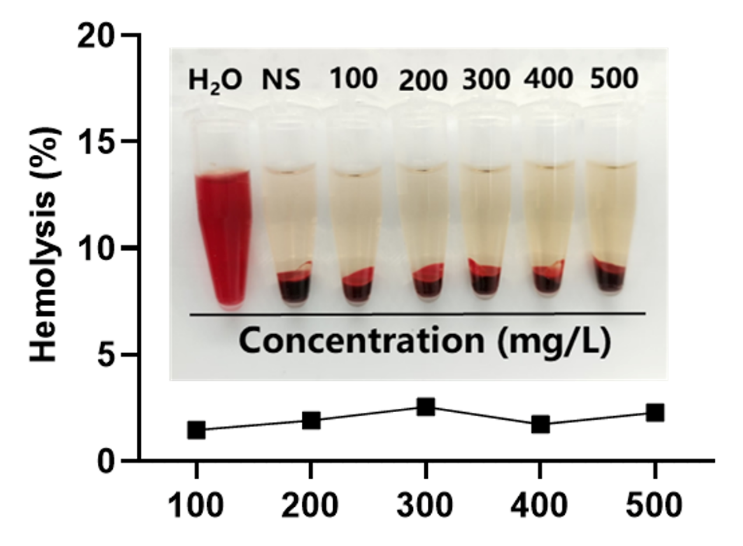


**Figure S11.** Hemolysis rate after incubation of red blood cells with different concentrations of Hf-rCDs (100, 200, 300, 400, and 500 mg/L).


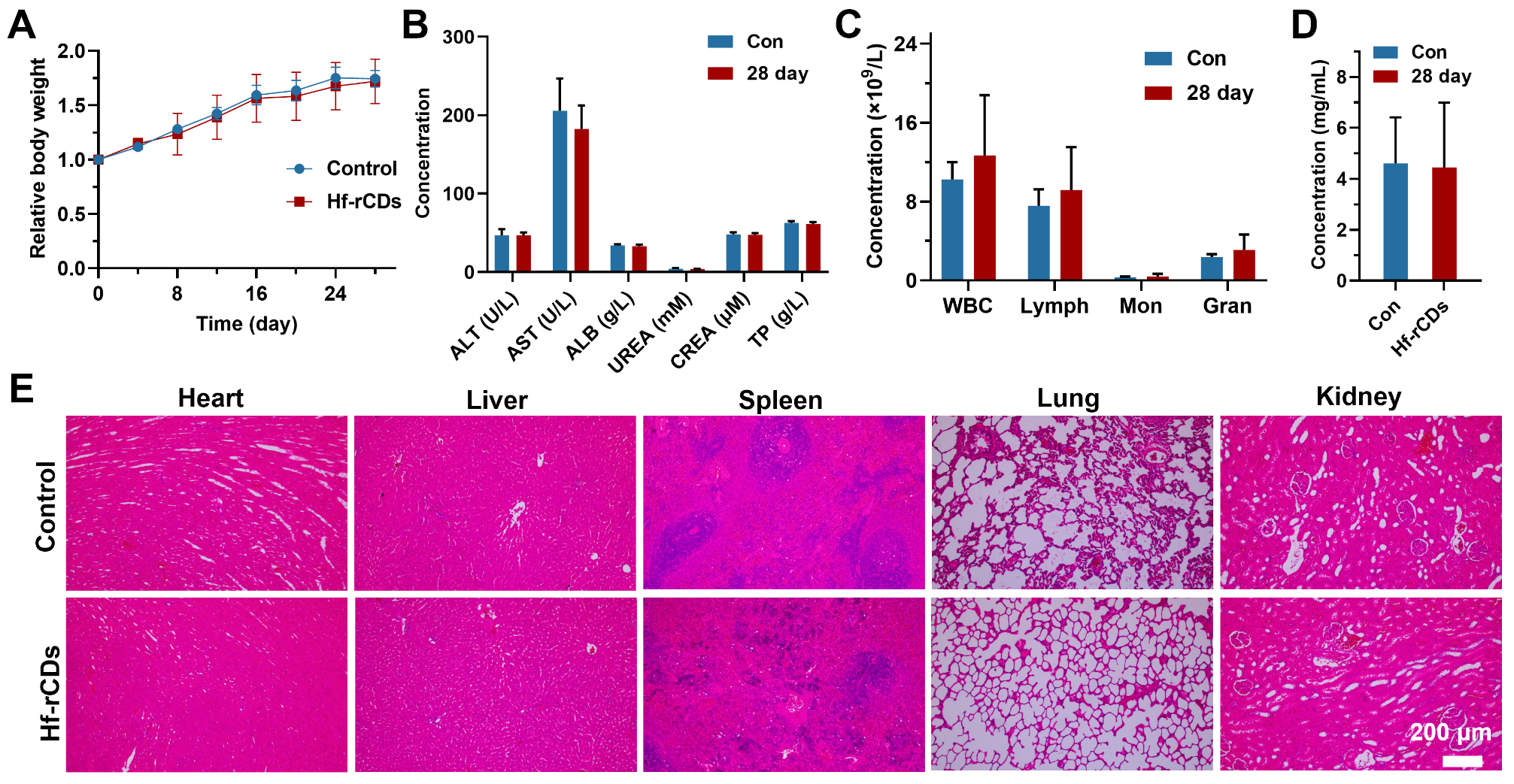


**Figure S12.** Body weight changes (A), biochemical analysis (B), complete blood count analysis (C) and IgG analysis (D) of rats with or without injection of Hf-rCDs (600 mg Hf-rCDs/kg) for 28 days (n = 3; mean ± SD). Histopathological examinations (E) of rats with or without injection of Hf-rCDs for 28 days. (600 mg Hf-rCDs/kg, n = 3). (ALT: alanine aminotransferase; AST: aspartate aminotransferase; ALB: albumin; UREA: blood urea nitrogen; CREA: creatinine; TP: total protein; WBC: white blood cells; Lymph: lymphocytes; Mon: monocytes; Gran: granulocytes)

**
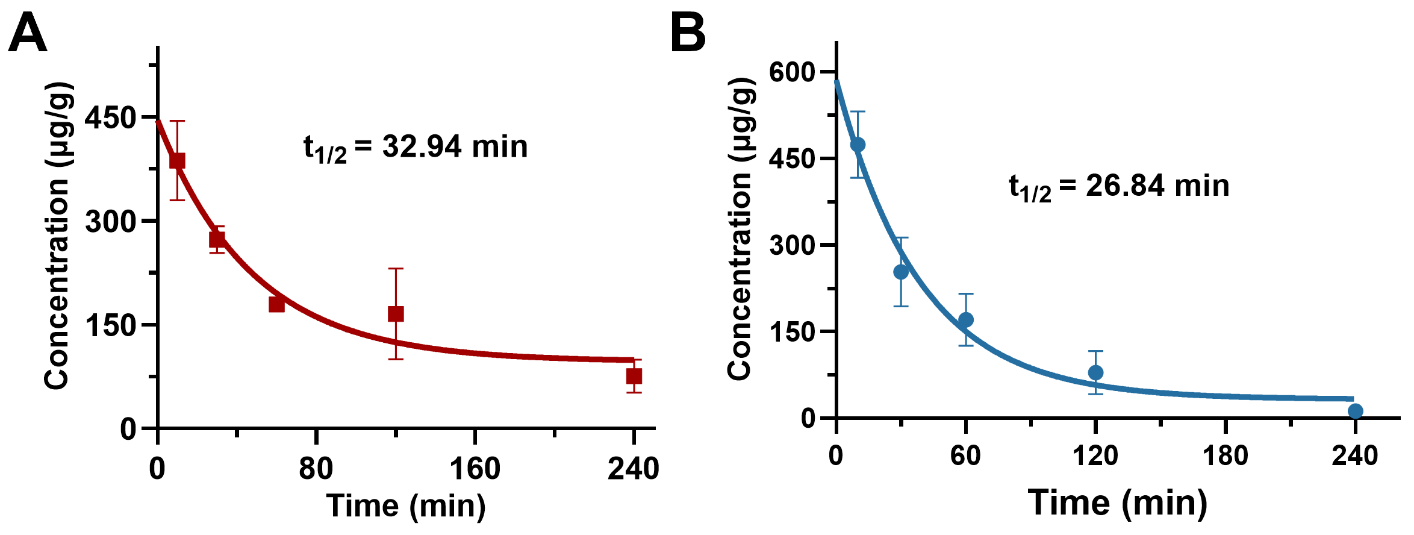
**

**Figure S13.** Blood half-life of Hf-rCDs (A) and iohexol (B) in rabbits (n = 3; mean ± SD).


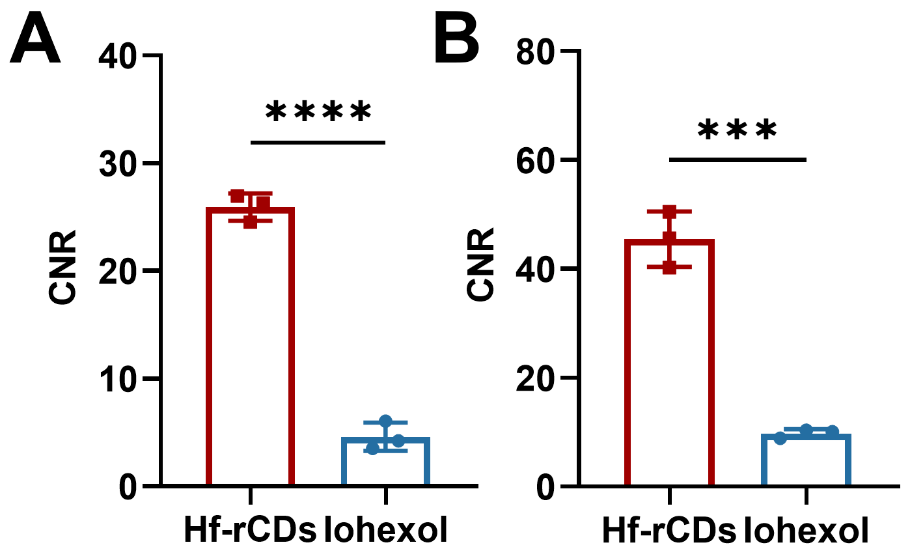


**Figure S14.** (A) contrast-to-noise ratio (CNR) of rabbit cervical vasculature after injection of Hf-rCDs or iohexol (n = 3 refers to three sections obtained from the same rabbit; mean ± SD; statistical analysis: Student's t-test, *****p* < 0.0001). (B) CNR of rabbit thoracoabdominal vasculature after injection of Hf-rCDs or iohexol (n = 3 refers to three sections obtained from the same rabbit; mean ± SD; statistical analysis: Student's t-test, ****p* < 0.001).

**
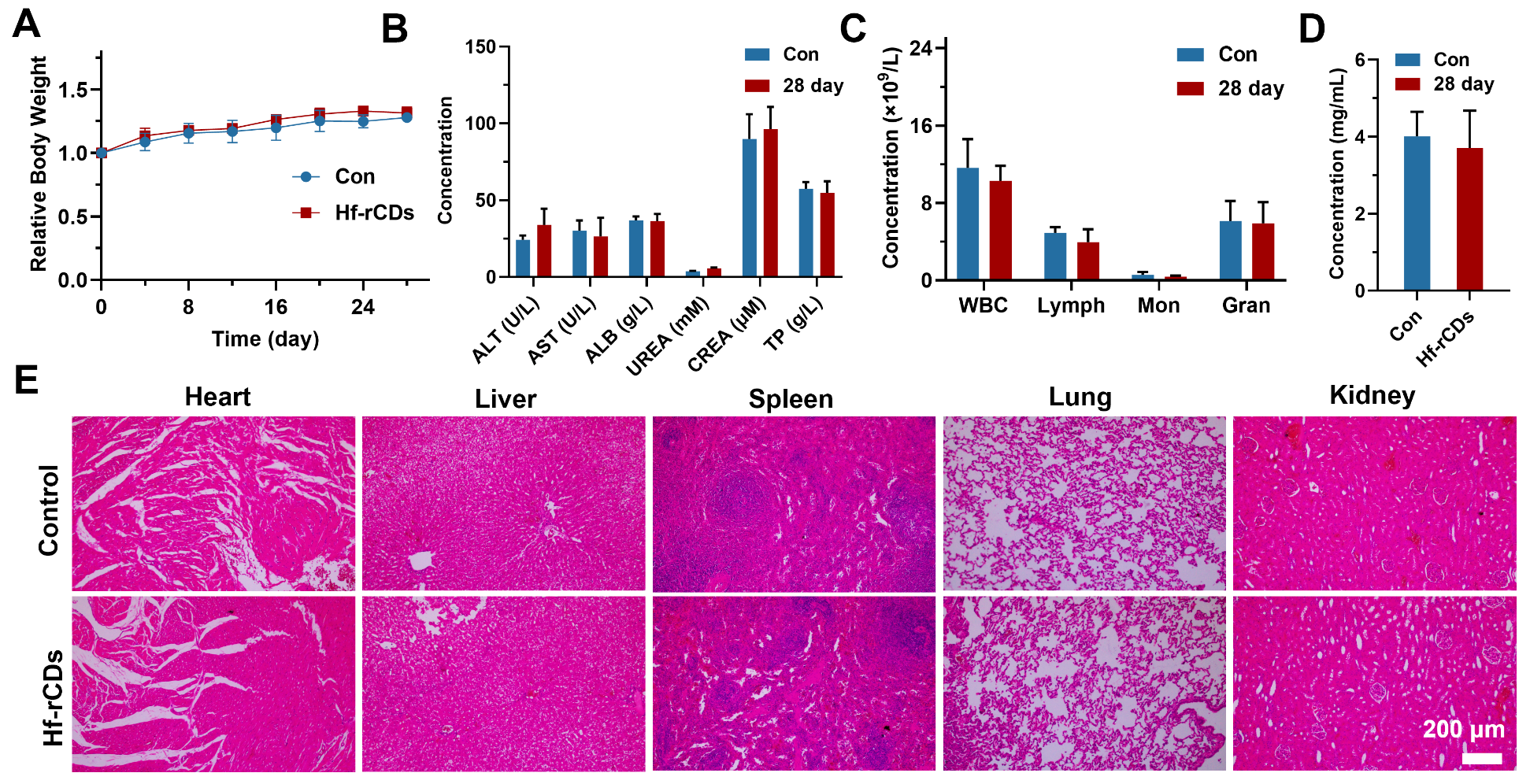
**

**Figure S15.** Body weight changes (A), biochemical analysis (B), complete blood count analysis (C) and IgG analysis (D) of rabbits with or without injection of Hf-rCDs (450 mg Hf-rCDs/kg) for 28 days (n = 3; mean ± SD). Histopathological examinations (E) of rabbits with or without injection of Hf-rCDs for 28 days. (450 mg Hf-rCDs/kg, n = 3). (ALT: alanine aminotransferase; AST: aspartate aminotransferase; ALB: albumin; UREA: blood urea nitrogen; CREA: creatinine; TP: total protein; WBC: white blood cells; Lymph: lymphocytes; Mon: monocytes; Gran: granulocytes)

**
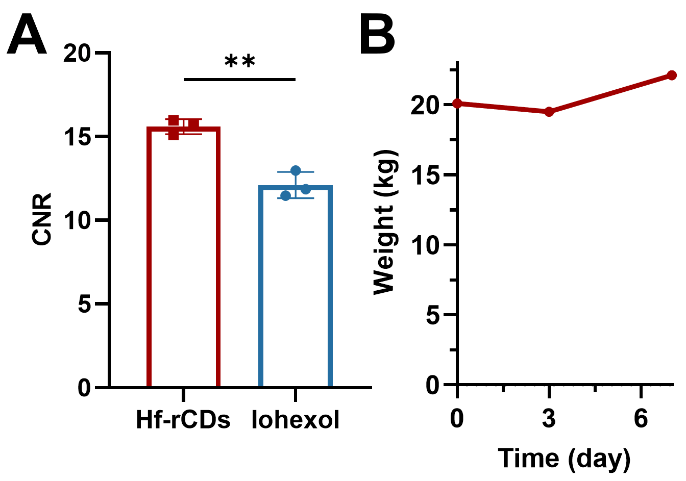
**

**Figure S16.** (A) CNR of swine cervical vasculature after injection of Hf-rCDs or iohexol (n = 3 refers to three sections obtained from the same swine; mean ± SD; statistical analysis: Student's t-test, ***p* < 0.01). (B) Body weight change curve of swine after injection of Hf-rCDs.

**Table S1**. The summary of representative Hf-based nanoprobes for *in vivo* CT imaging.

| Nanoprobe | Hf content | Gram scale production | Renal clearable | Animal species | Intravenous administration | Ref. |
| --- | --- | --- | --- | --- | --- | --- |
| Hf-rCDs | High (40.7%) | Yes  (2.6 g in lab) | Yes | rat, rabbit, swine | Yes | This work |
| Hf-CDs | 25.8% | No | Yes | mouse | Yes | [1] |
| Protein-HfO_2_ | - | - | Yes | mouse, rabbit | Yes | [2] |
| PEGylated HfO_2_ | 36% | - | No | mouse | Yes | [3] |
| Hf-UiO@SiO_2_@PEG | 46.4% | - | No | mouse | Yes | [4] |
| ZrCe-HCO | - | - | No | mouse | Yes (toxicity assessment)  No (tumor imaging/therapy) | [5] |
| PVP-HfO_2_ | 50.4% | Yes | No | rat | No | [6] |
| Antibody-HfO_2_ | - | - | No | rat | No | [7] |
| Nitrilotriacetic acid-HfO_2_ | - | - | No | rat | No | [8] |
| HfO_2_-IRDye 800CW | - | - | No | mouse | No | [9] |

Reference

[1] Y. Su, S. Liu, Y. Guan, Z. Xie, M. Zheng, X. Jing, *Biomaterials* **2020**, 255, 120110.

[2] D. Liu, F. Cao, Z. Xu, C. Zhao, Z. Liu, J. Pang, Z.-X. Liu, M. Moghiseh, A. Butler, S. Liang, W. Fan, J. Yang, *Adv. Mater.* **2024**, 36, 2308098.

[3] S. Li, H. Wu, L. Guo, X. Wang, G. Shu, X. Li, S.-K. Sun, *ACS Nano* **2025**, 19, 37266.

[4] K. E. deKrafft, W. S. Boyle, L. M. Burk, O. Z. Zhou, W. Lin, *J. Mater. Chem.* **2012**, 22, 18139.

[5] H. Shangguan, Q. Wang, S. Liu, C. Li, J. Qu, Y. Cui, Z. Tang, Y. Huang, N. Niu, J. Xu, *Nano Lett.* **2024**, 24, 11738.

[6] F. Ostadhossein, P. Moitra, N. Gunaseelan, M. Nelappana, C. Lowe, M. Moghiseh, A. Butler, N. de Ruiter, H. Mandalika, I. Tripathi, S. K. Misra, D. Pan, *Nanoscale Horiz.* **2022**, 7, 533.

[7] N. Gunaseelan, P. Moitra, P. Saha, T. Aditya, M. Moghiseh, K. Jonker, S. Gieseg, A. Butler, F. Kamal, D. Pan, *Adv. Sci.* **2024**, 11, 2408408.

[8] F. Ostadhossein, I. Tripathi, L. Benig, D. LoBato, M. Moghiseh, C. Lowe, A. Raja, A. Butler, R. Panta, M. Anjomrouz, A. Chernoglazov, D. Pan, *Adv. Funct. Mater.* **2020**, 30, 1904936.

[9] L. Deblock, B. Descamps, I. Goemaere, E. Goossens, G. Vergauwen, J. Debacker, P. Tummers, K. Remaut, I. Van Driessche, K. De Buysser, J. De Roo, C. Vanhove, *Chem. Mater.* **2023**, 35, 8883.
